# A rare coexistence of adrenal cavernous hemangioma with extramedullar hemopoietic tissue: a case report and brief review of the literature

**DOI:** 10.1186/1477-7819-7-13

**Published:** 2009-02-05

**Authors:** Nikolaos Arkadopoulos, Maria Kyriazi, Anneza I Yiallourou, Vaia K Stafyla, Theodosios Theodosopoulos, Nikolaos Dafnios, Vassilis Smyrniotis, Agathi Kondi-Pafiti

**Affiliations:** 1 2nd Department of Surgery, Aretaieion Hospital, Athens University School of Medicine, Athens, Greece; 2 Department of Pathology, Aretaieion Hospital, Athens University School of Medicine, Athens, Greece

## Abstract

**Background:**

Cavernous hemangiomas of the adrenal gland are rare, benign, non-functioning neoplastic tumors. To our knowledge, 55 cases have been reported in the literature to date.

**Case presentation:**

We report the first case of a large, non-functioning adrenal cavernous hemangioma that was incidentally found during the preoperative staging workup of a 75 year old woman with left breast adenocarcinoma. Imaging with US, CT scan and MRI showed a heterogeneous 8 cm mass with non-specific radiological features that was located on the left adrenal gland. The mass was surgically excised and pathology revealed an adrenal hemangioma with areas of extramedullar hemopoiesis.

**Conclusion:**

Although adrenal hemangiomas are rare and their preoperative diagnosis is difficult, they should always be included in the differential diagnosis of adrenal neoplasms.

## Background

Adrenals are an infrequent location for benign vascular tumors like cavernous hemangiomas-such tumors are most commonly situated on the skin or in the liver. Their clinical presentation is usually vague, with non-specific abdominal pain being the predominant symptom. Frequently, they are discovered as incidentalomas either during imaging or in autopsies. Since 1955, when Johnson and Jeppesen described the first adrenal cavernous hemangioma, only 55 cases have been reported in the literature [1]. We report a case of a large, non-functioning adrenal hemangioma that was found incidentally during pre-operative staging of a 75 year old woman with adenocarcinoma of the left breast.

## Case presentation

**Figure 1:**
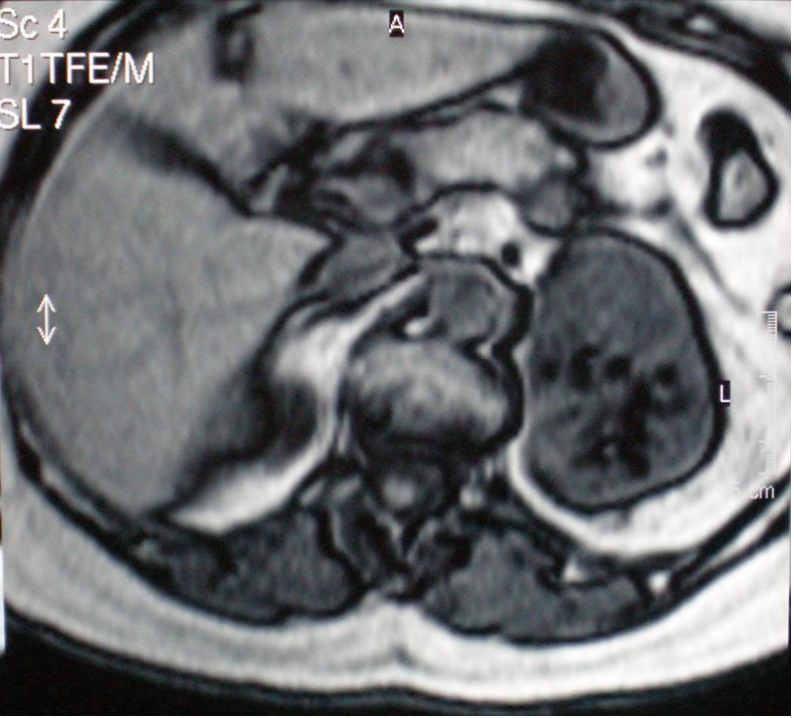
**MRI scan of a left adrenal hemangioma demonstrating hyperintensity on T1-weighted image with a fat component**.

**Figure 2:**
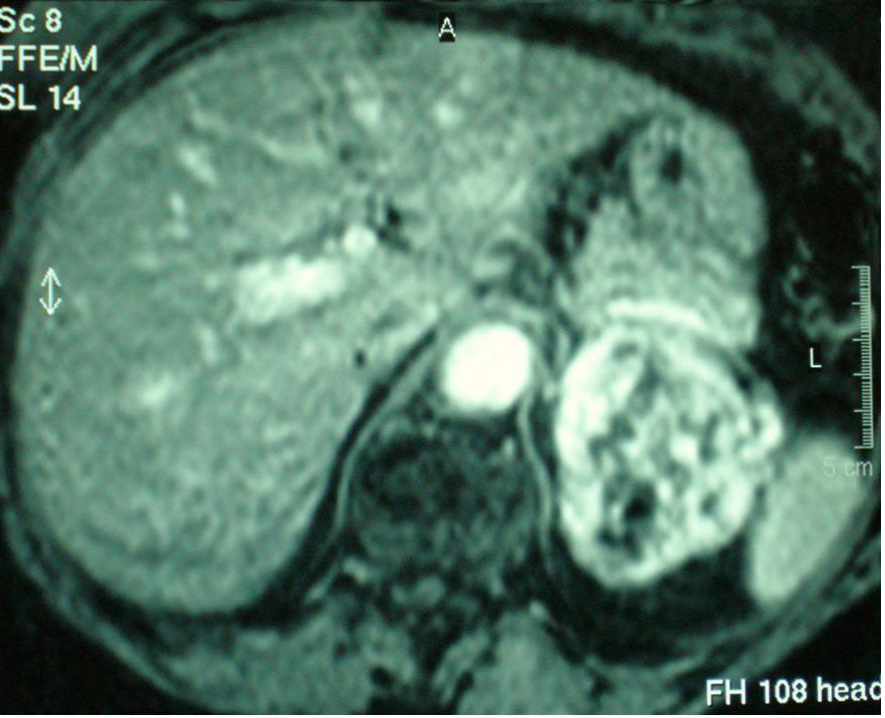
**MRI scan of a left adrenal hemangioma demonstrating hyperintensity on T2-weighted image and irregular peripheral enhancement**.

A 75 year old female patient with breast cancer was admitted to our hospital for surgical treatment. Her preoperative staging workup with an abdominal ultrasound, revealed a heterogeneous solid lesion of the left adrenal gland. Clinical examination and laboratory tests, including adrenal hormonal levels (plasma renin 7,40 pg/ml, plasma aldosterone 12,7 ng/dl, plasma adrenaline 27 pg/ml, plasma noradrenaline 243 pg/ml, 24 h urine metanephrine excretion 169 μg/24 h), were normal. Abdominal CT scan showed a well-defined, heterogeneous, retroperitoneal mass with speckled calcifications that measured 8 cm and was located on the left adrenal gland. After bolus IV injection of contrast medium the tumor showed irregular enhancement. On subsequent MRI, the tumor demonstrated hyperintensity on both T1- and T2-weighted images with fat component and irregular peripheral enhancement (Figure 1, 2). Malignancy could not be excluded due to the non-specific radiological features, therefore surgical resection was mandatory.

During the same operation, the patient underwent a left adrenalectomy through a left subcostal incision followed by modified radical left mastectomy. Her postoperative course was uneventful and she was discharged five days later.

**Figure 3:**
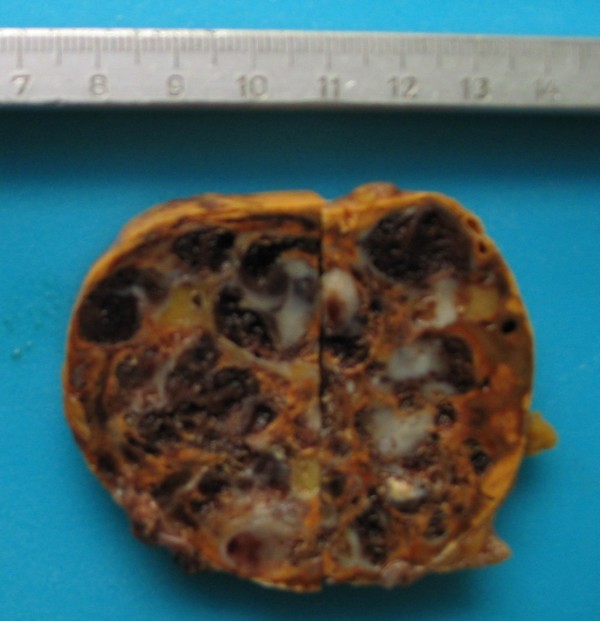
**Gross section of adrenal hemangioma showing macrocystic, haemorrhagic surface**.

On gross examination, the adrenal tumor appeared as a red tan mass measuring 8 cm × 6 cm × 4 cm. Focal red-purple hemorrhagic and cystic areas were present, along with diffuse calcifications. Normal adrenal gland parenchyma was noted on the surface of the mass (Figure 3).

**Figure 4:**
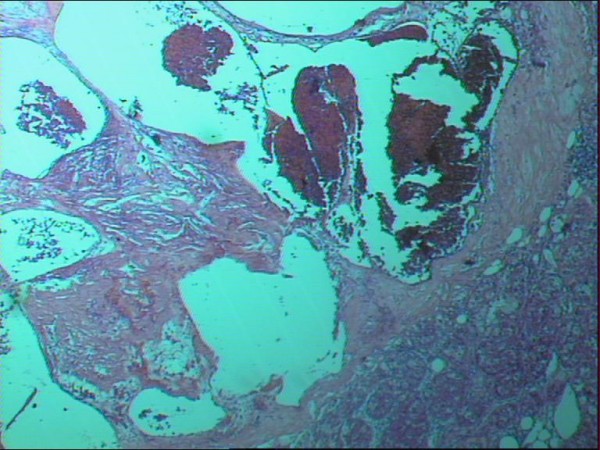
**Histological appearance of the adrenal hemangioma (hematoxylin-eosin × 25)**.

**Figure 5:**
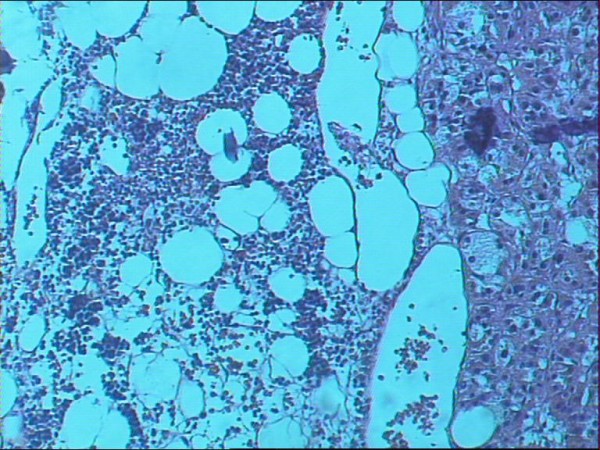
**Histological section of the adrenal hemangioma showing a focus of extramedullar hemopoiesis (hematoxylin-eosin × 25)**.

Microscopically, dilated, blood filled vascular spaces were observed. The spaces were lined by a single layer of thin endothelial cells with collagenous walls (Figure 4). Interestingly, areas of extramedullar hemopoiesis were also seen (Figure 5).

The histological diagnosis was that of an adrenal cavernous hemangioma with coexistence of extramedullar hemopoiesis and no signs of malignancy.

The pathology report on the breast specimen showed a grade II infiltrating tubular adenocarcinoma, measuring 5 cm in greatest diameter. None of the 13 excised lymph nodes had signs of malignancy.

## Discussion

The evolution of radiological imaging in the last 20 years resulted in increased detection rate of clinically inapparent adrenal masses, also known as adrenal incidentalomas. It is estimated that adrenal masses are an accidental finding in 1–5% of all abdominal CT scans performed. Adrenal hemangiomas, however, are extremely rare, and their differential diagnosis preoperatively is rather challenging.

Adrenal hemangiomas are most usually cavernous, unilateral lesions of the adrenal glands that appear in the sixth or seventh decade of life, with a 2:1 female to male ratio [2-4]. Their size ranges from 2 cm to 25 cm in diameter, with the majority measuring more than 10 cm [5-7]. They are most commonly non-functioning tumors, with only three cases of hormone-secreting adrenal hemangiomas being reported to date [8-10]. These unusual benign adrenal masses are usually detected as incidental radiological findings in abdominal imaging performed for various other reasons. They are hardly ever symptomatic, with abdominal pain due to mechanical mass effects on neighbouring structures being the main symptom. However, in two cases adrenal haemangiomas presented with spontaneous life-threatening retroperitoneal haemorrhage [3,11]. The adrenal glands are a common site of metastasis for various cancers, therefore adrenal masses must be excluded in the preoperative staging of several carcinomas (melanomas, lung, breast, renal and gastrointestinal cancers). Three cases of adrenal hemangiomas, coexisting with malignant tumors of other organs (non-small-cell lung cancer, common bile duct cancer and gynaecological cancer) [12-14] have been reported in the literature. This is the only case of adrenal hemangioma in a patient with breast cancer reported so far. Histologically, these tumors are primary mesenchymal vascular neoplasms with angioblastic cells predominating. Surprisingly, this is the only case reported with extramedullar hemopoietic tissue within a hemangioma.

Distinguishing a large adrenal hemangioma from other lesions of the adrenal glands, and especially from malignant tumors, can be very difficult. In most cases the final diagnosis is made by histopathology after surgical resection. However, there are some radiological features that, although not entirely specific, should raise the suspicion of adrenal haemangioma. CT scans usually display a characteristic peripheral patchy enhancement with progression to the centre of the tumor that is a common finding [15]. Speckled calcifications that appear throughout the mass are attributed to multiple phleboliths located in dilated vascular spaces [16,17]. Nonetheless, this is a common finding in other adrenal lesions, such as pheochromocytoma, carcinoma and adenoma, and cannot, therefore, be pathognomonic for hemangiomas.

MRI has been proven to be the best diagnostic tool so far. The most characteristic finding is the peripheral spotty and centripetal enhancement on dynamic studies. Marked hyperintensity on T2-weighted images in combination with focal hyperintensity in T1-weighted images, indicate areas of calcification and haemorrhage that are associated with adrenal hemangiomas [2,15,18]. Angiography usually reveals peripheral pooling of the contrast, persisting well during the venous phase [16,17].

The surgical indication for excision of the tumor is the size. Adrenal incidentalomas larger than 6 cm in diameter must be excised because the risk of adrenal cancer is 35% to 98%. For lesions measuring 4 cm to 6 cm, other imaging features, history of extra-adrenal malignancy, patient's preference, age and comorbitities should be taken into consideration. Adrenalectomy and follow-up with imaging are both acceptable in such cases [3]. Most adrenal hemangiomas reported so far have been treated surgically due to their size. Other indications for surgery include mass-effect type symptoms from neighbouring organs and complications, such as haemorrhage.

Adrenalectomy can be performed laparoscopically for lesions measuring less than 6 cm [7,19]. Larger tumors, that are technically challenging and more likely to be malignant are treated preferably with open technique through an anterior (subcostal or midline incision), posterior or thoracoabdominal approach.

## Conclusion

We presented a rare coexistence of an adrenal cavernous hemangioma with extramedullar hemopoietic tissue in a woman treated for breast cancer. Although rare, adrenal haemangioma should be included in the differential diagnosis of adrenal neoplasms. The main indication for surgical removal of an adrenal mass is its size. However, the risks of haemorrhage, necrosis and thrombosis necessitate surgical excision in most of the cases, especially for tumors more than 3 cm.

## Consent

Written informed consent was obtained from the patient for publication of this case report and any accompanying images. A copy of the written consent is available for review by the Editor-in-Chief of this journal.

## Competing interests

The authors declare that they have no competing interests.

## Authors' contributions

NA was responsible for critical revision of scientific content. MK drafted the manuscript. AIY participated in the design of the manuscript and helped to draft the manuscript. VKS contributed substantially to manuscript conception and design. TT assisted in the preparation of the manuscript.

ND participated in the acquisition of data and preparation of the manuscript. VS was the surgeon, approved the final version of the manuscript for publication. AKP performed histopathological and immunohistochemical analysis and contributed substantially to pathology content. All authors read and approved the final manuscript.
